# Association between energy‐adjusted dietary inflammatory index and total immunoglobulin E: A cross‐sectional study

**DOI:** 10.1002/fsn3.3854

**Published:** 2023-12-09

**Authors:** Liang Su, Fang Wang, Cheng Qin, Zhimin Wang, Xuesong Yang, Jianzhou Ye

**Affiliations:** ^1^ Department of Dermatology The First Affiliated Hospital of Yunnan University of Chinese Medicine Kunming China; ^2^ Department of Dermatology Yunnan Provincial Hospital of Traditional Chinese Medicine Kunming China; ^3^ The First Clinical School of Yunnan University of Chinese Medicine Kunming China

**Keywords:** body mass index, dietary inflammatory index, immunoglobulin E, obesity, the National Health and Nutrition Examination Survey

## Abstract

The relationship between a pro‐inflammatory diet, assessed by the dietary inflammatory index (DII), and allergic diseases has attracted attention. However, the association between DII and immunoglobulin E (IgE) remains uncertain. We aim to investigate the association between energy‐adjusted DII (E‐DII) and total IgE. We analyzed data from the 2005 to 2006 National Health and Nutrition Examination Survey. The relationship between E‐DII and total IgE was assessed using linear regression and logistic regression analysis. Meanwhile, we conducted a subgroup analysis stratified by body mass index (BMI) and analyzed the mediating role of BMI. We included 3614 adult participants. After controlling for confounding factors, there was no statistical association between E‐DII and total IgE (*β* 0.023, 95% CI −0.01 to 0.057, *p* = .173) and the risk of high total IgE (OR 1.036, 95% CI 0.977 to 1.099, *p* = .233). We conducted subgroup analysis stratified by BMI. After controlling for confounding factors, only in overweight groups, E‐DII was statistically associated with total IgE (*β* 0.076, 95% CI 0.017 to 0.135, *p* = .012) and the risk of high total IgE (OR 1.124, 95% CI 1.015 to 1.246, *p* = .025). Generalized additive models and smooth curve fittings showed a positive linear relationship between E‐DII and total IgE in overweight participants. No statistical association was noted for the mediation effect of BMI on the association between E‐DII and total IgE in the overweight group (*p* = .23). Overweight participants with higher E‐DII were potentially at risk of elevated total IgE.

## Graphical abstract

E‐DII was associated with total IgE levels in the overweight group. E‐DII was associated with the risk of high total IgE levels in the overweight group. The mediation effect of BMI was not significant in the overweight group.

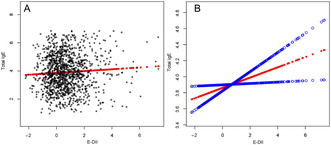

## 1 INTRODUCTION

Diet, an important component of lifestyle, is a modifiable behavioral risk factor for chronic disease development (Marx et al., 2021). Healthy dietary patterns are a global priority to decrease chronic diseases; however, unhealthy dietary patterns have been worsening in most parts of the world (Imamura et al., 2015). Consequently, dietary factors may remain the major driver of the global burden of chronic disease. Notably, the diet has been found to influence inflammation, as it can serve as a source of pro‐inflammatory compounds that can create and sustain a low‐grade inflammatory state (Garay‐Sevilla et al., 2021; Shivappa et al., 2017). Current evidence indicates that chronic low‐grade inflammation predisposes individuals to various chronic non‐communicable diseases (Calder et al., 2017; Hotamisligil, 2017). Different dietary patterns closely correlate with chronic inflammation, and the dietary inflammatory index (DII) has emerged as a widely recognized and effective tool for assessing the inflammatory potential of different diets (Shivappa et al., 2014). Numerous studies have demonstrated the close association between DII and a range of chronic diseases, such as hypertension and anxiety disorders (Torabynasab et al., 2023; Tyrovolas et al., 2017).

In chronic inflammatory states, abnormal activation of the immune system may increase the sensitivity and severity of allergic reactions (McCraw et al., 2021). The association between a pro‐inflammatory diet, as assessed by DII, and allergic diseases has attracted attention. However, the results are not entirely consistent (Han et al., 2018; Schutte et al., 2022). Indeed, the etiological mechanisms of allergic diseases are complex, and immunoglobulin E (IgE), an antibody within the immune system, plays a central role in the pathogenesis of allergic inflammation (Guntern & Eggel, 2020). Allergic diseases can be categorized into two groups, namely IgE‐mediated and non‐IgE‐mediated (Wang, Li, et al., 2022). IgE‐mediated allergy is the most frequent hypersensitivity disorder, which affects over 30% of the population (Shamji et al., 2021). An increase in total IgE levels can be observed in many patients with allergic diseases such as atopic dermatitis, asthma, and allergic rhinitis (Shamji et al., 2021). However, the relationship between energy‐adjusted DII (E‐DII) and total IgE levels lacks sufficient evidence.

Our study aims to investigate the association between E‐DII and total IgE, which may provide insights into the association between pro‐inflammatory diets and allergic diseases. Additionally, considering the evidence linking obesity to elevated levels of total IgE (Carballo et al., 2021), we will conduct a subgroup analysis stratified by body mass index (BMI) and analyze the mediating role of BMI.

## 2 METHODS

### 2.1 Study design and participants

The National Health and Nutrition Examination Survey (NHANES) is a serial cross‐sectional survey that collects data on the nutritional and health status of the noninstitutionalized US population (Ostojic et al., 2023; Sun et al., 2023). This nationwide survey provides data through questionnaires, physical examinations, and laboratory tests (Ahluwalia et al., 2016; Shen et al., 2023). The investigation protocol was approved by National Center for Health Statistics Ethics Review Board and each participant signed the written informed consent (Santos & Dhamoon, 2020).

The 2005–2006 NHANES data was used for this analysis. We included participants aged 20 years or older with available information on dietary data and total IgE. We excluded participants who had IgE deficiency (IgE < 2.5 kU/L) and very high IgE levels (IgE ≥ 1000 kU/L) (Ferastraoaru & Rosenstreich, 2018). Additionally, individuals with extreme energy intake (<500 or > 5000 kcal/day for females and 500 or > 8000 kcal/day for males) were excluded (Sun et al., 2023). And we excluded participants with missing data on covariates.

### 2.2 Measurements

#### 2.2.1 E‐DII

The DII was utilized to assess the potential inflammatory levels of dietary components, and its calculation and validation have been described in detail elsewhere (Chen et al., 2022; Sun et al., 2023). In this study, DII was calculated based on dietary intake data collected from the 24 h dietary recalls in the NHANES database. Available food parameters for DII calculating included: vitamins A, B1, B2, B6, B12, C, and E; carbohydrates; alcohol; caffeine; fiber; saturated fatty acids; monounsaturated fatty acids; polyunsaturated fatty acids; cholesterol; omega‐3 fatty acids; omega‐6 fatty acids; niacin; beta‐carotene; iron; magnesium; folic acid; zinc; selenium; protein; total fat; and energy. To minimize the effect of total energy intake, the DII was calculated per 1000 kcal of food consumed (E‐DII) (Fu et al., 2021; Huang et al., 2022). The final E‐DII is a continuous score, with higher values indicating more pro‐inflammatory diets.

#### 2.2.2 Total IgE

Serum samples were collected at the NHANES examination site and analyzed in the NHANES laboratory, and serum total IgE was tested using the Pharmacia Diagnostics ImmunoCAP 1000 System (Kalamazoo, MI, USA) (Min & Min, 2019; Xi et al., 2023). Total IgE was analyzed as a continuous variable, while participants were categorized into two groups according to total IgE levels: normal total IgE levels (2.5–100 kU/L) and high total IgE levels (100–1000 kU/L), with the latter indicating total IgE sensitization (Ferastraoaru & Rosenstreich, 2018; Xi et al., 2023).

#### 2.2.3 Study covariates

Considering that age, gender, race, BMI, alcohol consumption, smoking, diabetes, hypertension, and physical exercise may affect E‐DII and total IgE, these variables were included in multivariable models (Min & Min, 2019; Sun et al., 2023; Xi et al., 2023). Smoking, alcohol consumption, and physical exercise were determined based on responses to the relevant questions. Diabetes and hypertension were confirmed through self‐reported clinician diagnosis. In the subgroup analysis stratified by BMI, participants were categorized into three categories: normal weight (<25 kg/m^2^), overweight (25–29.9 kg/m^2^), and obesity (≥30 kg/m^2^) (Reeder et al., 2022).

#### 2.2.4 Statistical analysis

Statistical analysis utilized the complex sampling weight following the NHANES analytic guidance. We expressed continuous variables as the mean ± standard deviation or median values (interquartile ranges) and a *t*‐test or Kruskal–Wallis test was used to compare the difference between them. For categorical variables, proportions were expressed and a chi‐square test was applied to assess the difference between them. We utilized multivariable regression to explore the association between E‐DII and total IgE, including the unadjusted Model I, minimally adjusted Model II (adjusted for gender, age, race), and fully adjusted Model III (adjusted for gender, age, race, BMI, alcohol consumption, smoking, diabetes, hypertension, and physical exercise). Since the distribution of total IgE was skewed, a natural logarithmic transformation was performed (Wells et al., 2014). Statistical analyses were performed by EmpowerStats software (http://www.empowerstats.com) and R version 4.1.1 (http://www.R‐project.org, The R Foundation). Statistical significance was set at *p* < .05.

## 3 RESULTS

### 3.1 Characteristics of participants

A total of 3614 participants were included in our analysis. The flowchart is presented in Figure S1. The clinical characteristics of the participants based on total IgE levels are represented in Table 1, including 2532 participants with normal total IgE levels and 1082 participants with high total IgE levels. Between the normal total IgE group and high total IgE group, there were significant differences in baseline data on BMI, gender, race, diabetes, and physical exercise (*p* < .05), with no significant differences in baseline data on age, E‐DII, alcohol consumption, smoking, and hypertension.

**TABLE 1:** Participant characteristics according to total IgE levels.

Characteristic	Total	Normal total IgE levels	High total IgE levels	p‐value
Number of subjects	3614	2532	1082	
Age (years)	45.5 (31)	46 (30)	45 (32)	.069
BMI (kg/m^2^)	27.66 (7.53)	27.41 (7.51)	28.19 (7.6)	.002
E‐DII	0.55 (1.51)	0.55 (1.55)	0.54 (1.46)	.995
Gender				<.001
Male	1738 (48.09%)	1114 (44.00%)	624 (57.67%)	
Female	1876 (51.91%)	1418 (56.00%)	458 (42.33%)	
Race				<.001
Mexican American	725 (20.06%)	458 (18.09%)	267 (24.68%)	
Other hispanic	110 (3.04%)	71 (2.80%)	39 (3.60%)	
Non‐hispanic white	1875 (51.88%)	1450 (57.27%)	425 (39.28%)	
Non‐hispanic black	771 (21.33%)	465 (18.36%)	306 (28.28%)	
Other race–including multi‐racial	133 (3.68%)	88 (3.48%)	45 (4.16%)	
Alcohol				.369
Yes	2497 (69.09%)	1738 (68.64%)	759 (70.15%)	
No	1117 (30.91%)	794 (31.36%)	323 (29.85%)	
Smoking				.43
Yes	1724 (47.70%)	1197 (47.27%)	527 (48.71%)	
No	1890 (52.30%)	1335 (52.73%)	555 (51.29%)	
Diabetes				.028
Yes	351 (9.71%)	228 (9.00%)	123 (11.37%)	
No	3263 (90.29%)	2304 (91.00%)	959 (88.63%)	
Hypertension				.357
Yes	1100 (30.44%)	759 (29.98%)	341 (31.52%)	
No	2514 (69.56%)	1773 (70.02%)	741 (68.48%)	
Physical				<.001
Inactive	2025 (56.03%)	1466 (57.90%)	559 (51.66%)	
Active	1589 (43.97%)	1066 (42.10%)	523 (48.34%)	

*Note*: Data are presented as medians (interquartile ranges) or number of subjects (percentage).

### 3.2 Association of E‐DII with total IgE

The linear regression was employed to estimate the correlation between E‐DII and total IgE levels, and the results are shown in Table 2. The association was not significant in Model I (*β* −0.003, 95% CI −0.036 to 0.031, *p* = .878), Model II (*β* 0.031, 95% CI −0.003 to 0.064, *p* = .071), and Model III (*β* 0.023, 95% CI −0.01 to 0.057, *p* = .173). The logistic regression model was applied to assess the correlation between DII and the risk of high total IgE levels, and the results are shown in Table 3, which are consistent with linear regression. The association was not significant in Model I (OR 1.014, 95% CI 0.96 to 1.071, *p* = .619), Model II (OR 1.044, 95% CI 0.986 to 1.107, *p* = .142), and Model III (OR 1.036, 95% CI 0.977 to 1.099, *p* = .233).

**TABLE 2:** Association between E‐DII and total IgE levels.

Exposure	Model I	Model II	Model III
E‐DII	−.003 (−0.036, 0.031) .878	.031 (−0.003, 0.064) .071	.023 (−0.01, 0.057) .173

*Note*: Data are *β* (95% CI) and *p*‐value. Model I: adjusted for: none. Model II: adjust for: gender, age, race. Model III: gender, age, race, BMI, alcohol consumption, smoking, diabetes, hypertension, and physical exercise.

**TABLE 3:** Association between E‐DII and the risk of high total IgE levels.

Exposure	Model I	Model II	Model III
E‐DII	1.014 (0.96, 1.071) .619	1.044 (0.986, 1.107) .142	1.036 (0.977, 1.099) .233

*Note*: Data are OR (95% CI) and *p*‐value. Model I: adjusted for: none. Model II: adjusted for: gender, age, race. Model III: gender, age, race, BMI, alcohol consumption, smoking, diabetes, hypertension, and physical exercise.

### 3.3 Subgroup analysis stratified by BMI

We conducted subgroup analyses stratified by BMI, and Table 4 shows the results of the linear regression. Among the overweight groups, there was no statistical association between E‐DII and total IgE levels in the unadjusted model (*β* 0.049, 95% CI −0.008 to 0.106, *p* = .093), while there was a statistical association in the fully adjusted model (*β* 0.076, 95% CI 0.017 to 0.135, *p* = .012). No statistical association was noted in the normal weight group and obesity group. Table 5 shows the results of the logistic regression, which are consistent with linear regression. Among the overweight groups, there was no statistical association between DII and the risk of high total IgE levels in the unadjusted model (OR 1.066, 95% CI 0.972 to 1.17, *p* = .175), while there was a statistical association in the fully adjusted model (OR 1.124, 95% CI 1.015 to 1.246, *p* = .025). No statistical association was noted in the normal weight group and obesity group. We applied generalized additive models and smooth curve fittings to evaluate the associations between E‐DII and total IgE levels in overweight participants (Figure S2). There was a linear relationship between E‐DII and total IgE levels in overweight participants, with total IgE levels increasing as E‐DII scores increased.

**TABLE 4:** Subgroup analysis of the association between E‐DII and total IgE levels stratified by BMI.

	Number	Unadjusted model	Adjusted model
Normal weight	1087	−.029 (−0.088, 0.03) .333	.003 (−0.056, 0.063) .911
Overweight	1274	.049 (−0.008, 0.106) .093	.076 (0.017, 0.135) .012
Obesity	1253	−.02 (−0.078, 0.037) .484	.012 (−0.045, 0.069) .682

*Note*: Data are *β* (95% CI) and *p*‐value. Adjusted model: gender, age, race, alcohol consumption, smoking, diabetes, hypertension, and physical exercise.

**TABLE 5:** Subgroup analysis of the association between E‐DII and the risk of high total IgE levels stratified by BMI.

	Number	Unadjusted model	Adjusted model
Normal weight	1087	1.003 (0.907, 1.109) .959	1.04 (0.932, 1.162) .483
Overweight	1274	1.066 (0.972, 1.17) .175	1.124 (1.015, 1.246) .025
Obesity	1253	0.968 (0.883, 1.061) .488	0.978 (0.887, 1.079) .664

*Note*: Data are OR (95% CI) and *p*‐value. Adjusted model: gender, age, race, alcohol consumption, smoking, diabetes, hypertension, and physical exercise.

### 3.4 Mediation analysis of BMI in overweight participants

We explored the mediation effect of BMI on the association between E‐DII and total IgE levels in the overweight group, as shown in Table 6. The direct effect was significant (*p* = .008), while the mediation effect was not significant (*p* = .23).

**TABLE 6:** Mediation analysis of BMI in overweight participants.

	Estimate	95% CI lower	95% CI upper	*p*‐value
Total effect	0.109	0.037	0.228	.012
Mediation effect	−0.002	−0.014	0.003	.23
Direct effect	0.111	0.041	0.231	.008
Proportion mediated	−0.02	−0.17	0.032	.242

*Note*: Gender, age, race, alcohol consumption, smoking, diabetes, hypertension, and physical exercise were adjusted.

## 4 DISCUSSION

With this study including 3614 participants from the NHANES 2005–2006, we investigated the association between E‐DII and total IgE levels. Our study found a statistical association between E‐DII scores and total IgE levels, as well as the risk of high total IgE levels, in overweight individuals. However, no statistical association was observed in the normal weight group and the obesity group. Generalized additive models and smooth curve fittings showed a positive linear relationship between E‐DII and total IgE levels in overweight participants. Additionally, we conducted the mediation effect of BMI on the association between E‐DII and total IgE levels in the overweight group, while the mediation effect was not significant. Our findings revealed that overweight participants with higher E‐DII scores were potentially at risk of elevated total IgE levels.

Diet may affect the development of allergic disease in multiple ways, such as a potential source of allergens or providing substrates for components that interfere with allergic pathology, as well as modulating inflammation and immune responses (Hogenkamp et al., 2020; Lopez‐Fandino, 2020). A cohort study of nutritional patterns and allergic disease development in 10‐year‐old children reported that children with atopic dermatitis exhibited some changes in their intake of vitamins, sugars, and fiber. Meanwhile, it was more likely to show a less anti‐inflammatory/more pro‐inflammatory dietary pattern (Schutte et al., 2022). Actually, in recent years, the focus has shifted towards recognizing the significance of the overall diet pattern rather than the consumption of individual nutrients or foods in isolation (Wang, Liao, et al., 2022). It has been established that dietary patterns have the ability to regulate inflammation within the body (Salas‐Salvado et al., 2008). DII and logically expanded E‐DII were utilized to evaluate the relationship between various nutrients as well as food and underlying inflammation (Wang, Liao, et al., 2022). Higher DII and E‐DII were associated with elevated levels of inflammatory markers in the bloodstream, including C‐reactive protein, interleukin‐6, fibrinogen, and white blood cell counts (Chen et al., 2021). IgE plays a central role in the pathogenesis of allergic inflammation (Guntern & Eggel, 2020). Our findings revealed that overweight participants with higher E‐DII scores were potentially at risk of increased total IgE levels, implying that a pro‐inflammatory diet may heighten the susceptibility to allergic diseases. Similarly, previous research reported that a high DII represents a higher risk of allergic rhinitis, while a high‐vegetable diet characterized by a high intake of anti‐inflammatory nutrients resulted in a lower risk (Oh et al., 2020). Our findings emphasize the overall impact of dietary patterns on allergic diseases and remind overweight individuals that they may need to pay more attention to adjusting dietary patterns to reduce the risk of allergic diseases.

Dietary components play a critical role in developing and maintaining optimal immune system function, such as zinc, vitamin D, and other nutritional factors that can impact the nature of the immune response, and ensure proper immune system functioning (Mazzocchi et al., 2017). An animal experiment found that vitamin D deficiency contributed to the severity of asthma, with worsening eosinophilic inflammation and airway remodeling, and that dietary supplementation with vitamin D improved both of these pathological abnormalities and resulted in a significant reduction in total IgE levels (Vasiliou et al., 2014). Similarly, recent evidence reveals that vitamin A availability determines susceptibility to allergic diseases and that allergic patients typically exhibit reduced serum vitamin A levels, with potential explanations related to interference with inflammatory signaling pathways by vitamin A and its active metabolites (Hufnagl & Jensen‐Jarolim, 2019). Meanwhile, besides the pro‐inflammatory or anti‐inflammatory effects on innate immune cells, lipids in dietary components can impact antigen presentation to adaptive immune cells, modify the immunostimulatory properties of proteins, alter the digestibility and intestinal absorption of proteins, and change allergen bioavailability (Lopez‐Fandino, 2020). In particular, n‐3 long‐chain polyunsaturated fatty acids may possess immunomodulatory properties to reduce unwanted inflammation and decrease the risk of allergy development (Hogenkamp et al., 2020). Moreover, diet plays a key role in maintaining gut microbiota diversity and the subsequent composition of microbial metabolites (Zheng et al., 2020). A diet with high DII potentially mediates alterations in gut microbiota and metabolite, which may induce T helper 2 cell differentiation and IgE isotype switching (Salameh et al., 2020; Tian et al., 2022). Thus, microbiota dysbiosis, mediated by high DII, may be an additional possible explanation for our results. In subgroup analyses, our results showed E‐DII was not statistically associated with total IgE levels in the normal weight group and the obese group. Actually, obesity is recognized as a low‐grade chronic inflammatory state (Lopez‐Fandino, 2020). A previous study investigating lifestyle factors and inflammation found that statin use was associated with lower high‐sensitivity C‐reactive protein in the overweight populations, but this association was not statistically significant in the obese populations (Kantor et al., 2013). This result may be attributed to the fact that obese individuals have much higher levels of inflammation, making reduction of inflammation relatively intractable (Kantor et al., 2013). Similarly, the much higher level of inflammation in obese individuals may make the effect of pro‐inflammatory diet on increased inflammation relatively difficult. Notably, obesity is also often associated with high total IgE levels (Carballo et al., 2021). Hence, it is tempting to speculate that the effects of pro‐inflammatory diet on total IgE levels may be limited in the obesity group. In addition, obesity has been reported to potentially alter the metabolism of polyunsaturated fatty acid (an important component in DII calculations) through various pathways that are not solely attributable to altered dietary polyunsaturated fatty acid intake (Pickens et al., 2017). Hence, another explanation may be related to the effect of obesity on the metabolism of dietary components. Regarding the absence of a statistical association in the normal weight group, it is possible that normal weight individuals are more tolerant to the adverse effects of pro‐inflammatory diet.

To our knowledge, this is the first study to assess the association between E‐DII scores and total IgE levels. The strength of this study is that we included a large sample of participants, and statistical analysis utilized the complex sampling weight, which contributed to the external validity of our results. Considering the global prevalence of obesity, another study strength is that we conduct a subgroup analysis stratified by BMI and analyze the mediating role of BMI. However, there are also some limitations in our study that need to be noticed. Firstly, due to the design of the cross‐sectional study, no causal or temporal relationship can be established between E‐DII and total IgE levels. Secondly, similar to the majority of previous studies, because of limited data, we cannot adjust for other covariates such as atopic dermatitis, food allergy, asthma, and atherosclerosis. Consequently, our study cannot deny the effect of other possible confounding factors. Thirdly, further stratification of obesity contributes to extending our results. However, the limited sample size limited our further analyses. Lastly, dietary intakes are self‐reported, which may carry a reporting bias. Further well‐designed controlled studies are needed to elucidate the association between E‐DII scores and total IgE levels.

## 5 CONCLUSIONS

Our results revealed that overweight participants with higher E‐DII scores were potentially at risk of elevated total IgE levels. IgE plays a central role in the pathogenesis of allergic inflammation. Our findings emphasized the overall impact of dietary patterns on allergic diseases and remind overweight individuals that they may need to pay more attention to adjusting dietary patterns to mitigate the risk of allergic diseases.

## AUTHOR CONTRIBUTIONS

**Liang Su:** Conceptualization (equal); data curation (lead); formal analysis (lead); writing – original draft (equal); writing – review and editing (equal). **Fang Wang:** Writing – original draft (equal); writing – review and editing (equal). **Cheng Qin:** Writing – review and editing (equal). **Zhimin Wang:** Writing – review and editing (equal). **Xuesong Yang:** Conceptualization (equal); supervision (equal). **Jianzhou Ye:** Conceptualization (equal); supervision (equal).

## FUNDING INFORMATION

This work was supported by the National Natural Science Foundation of China (No. 82260940), Yunnan Provincial Department of Education Science Research Fund Project (No. 2023Y0469), and High‐level key discipline construction project of National Administration of Traditional Chinese Medicine, and Health Commission of Yunnan Province‐Scientific and Technological Talents and Platform Program Academician (Expert) Workstation Project (No.202005AF150075).

## CONFLICT OF INTEREST STATEMENT

The authors report no conflicts of interest.

## ETHICS STATEMENT

The survey protocol was approved by the National Center for Health Statistics Ethics Review Board.

## INFORMED CONSENT STATEMENT

All study participants submitted written informed consent.

## Supporting information


Figure S1.

## Data Availability

All data in the current analysis are publicly available on the NHANES website.
