# Supplementary material for: Association between energy‐adjusted dietary inflammatory index and total immunoglobulin E: A cross‐sectional study
Source: Food Sci Nutr. 2023 Dec 9;12(3):1627–34. doi: 10.1002/fsn3.3854 (PMC10916634; doi:10.1002/fsn3.3854)
Supplement: Supplementary file 1 — Figure S1. [file FSN3-12-1627-s001.docx]

**Association between energy-adjusted dietary inflammatory index and total immunoglobulin E: A cross-sectional study**

Liang Su et al.

**“Online Supplementary Material”**


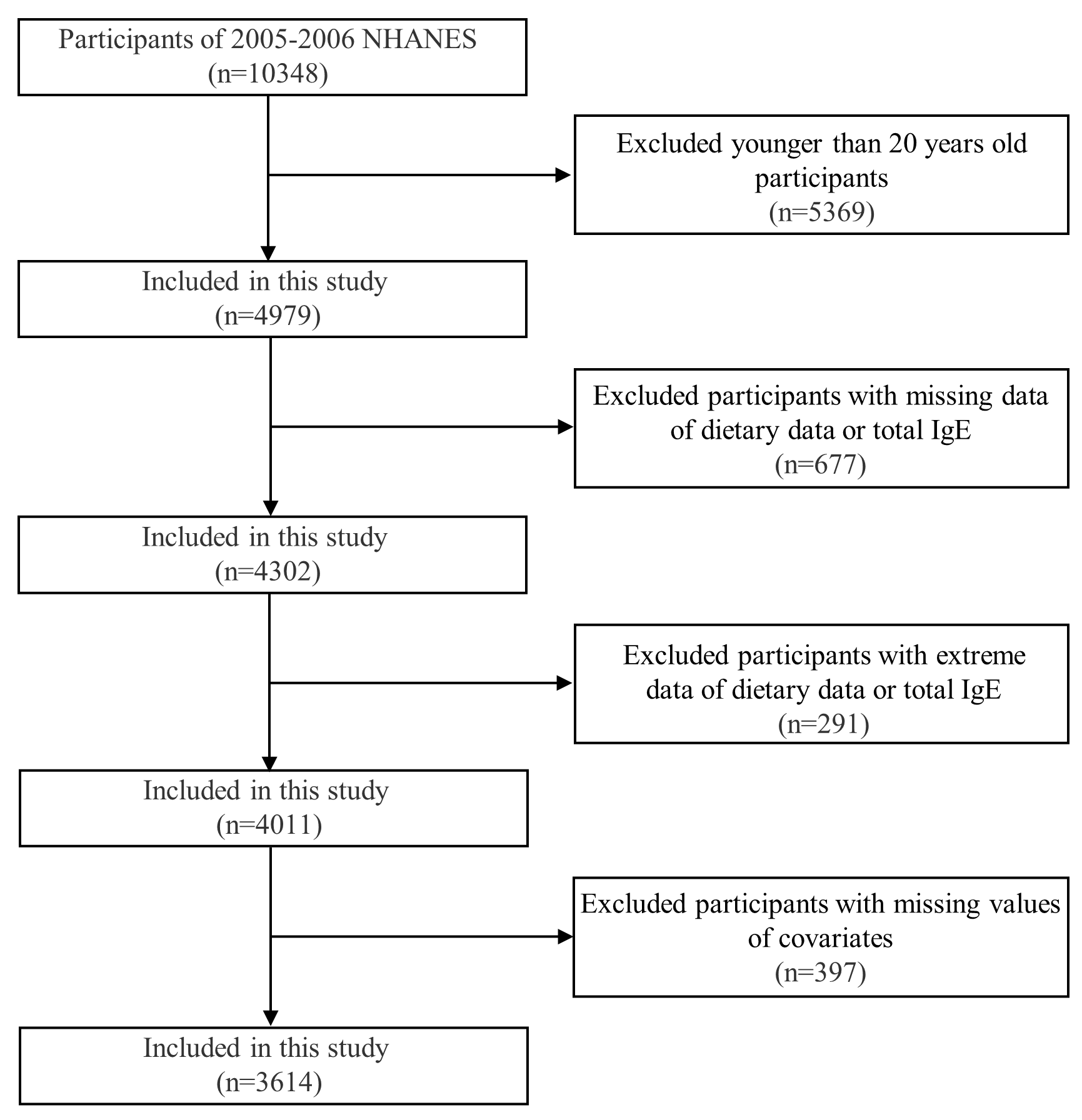


Supplemental Figure 1: Flow chart population included in our analysis.


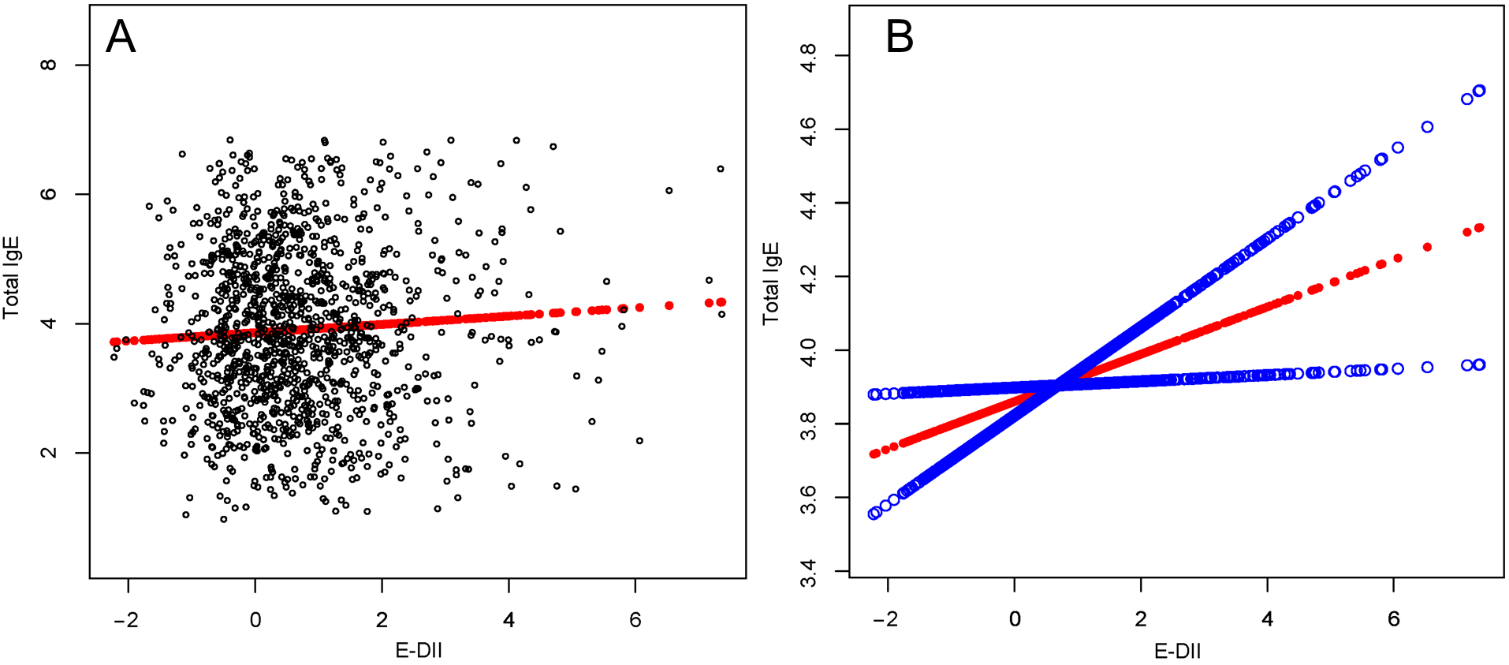


Supplemental Figure 2: The association between energy-adjusted dietary inflammatory index (E-DII) and immunoglobulin E (IgE) in the overweight participants. (A) Each black point represents a sample. (B) Solid red line represents the smooth curve fit between variables. Blue lines represent the 95% of confidence interval from the fit. Gender, age, race, alcohol consumption, smoking, diabetes, hypertension, and physical exercise were adjusted.
